# Intranasal Esketamine + CBT: a 6 months follow-up of a resistant depression complicated case

**DOI:** 10.1192/j.eurpsy.2022.1431

**Published:** 2022-09-01

**Authors:** V. Martiadis, F. Raffone, R. Cerlino, F. Mistico, M. Russo

**Affiliations:** ASL Napoli 1 Centro, Department Of Mental Health, Napoli, Italy

**Keywords:** esketamine, treatment resistant depression, CBT, Quality of Life

## Abstract

**Introduction:**

TRD is a highly disabling condition, often responsible for chronic clinical course, high number of relapses and elevated suicide risk. Intranasal esketamine is currently the only available pharmacological therapy specifically indicated for TRD, as add-on therapy to antidepressant treatment with SSRI or SNRI.

**Objectives:**

The purpose of the study was to evaluate the safety and efficacy of intranasal esketamine associated with CBT in a complex clinical case of TRD, over a six-month follow-up.

**Methods:**

A 67-year-old patient with TRD was selected for treatment with intranasal esketamine+CBT as add-on to antidepressant therapy. Before each treatment session the HAM-D rating scale was administered. The patient underwent weekly CBT sessions throughout the 6 months follow-up. The effect on physical well-being and social functioning was evaluated by means of Short-Form-Health-Survey-36.

**Results:**

After the first two administrations of intranasal esketamine the total score on HAM-D decreased by 10 units (from 26 to 16). After 6 weeks of treatment decreased from 26 to 12 with the disappearance of suicidal ideation present at T0. After 6 months the total HAM-D score decreased from 26 to 8. Treatment was well tolerated, with mild adverse effects, confined to the first two hours post-administration. In particular, mild sedation, dizziness, slight transient blood pressure rise were reported, never required medical intervention and resolved spontaneously during the observation period.

**Conclusions:**

Intranasal esketamine add-on therapy + CBT was an effective and safe treatment allowing to achieve and maintain symptomatic remission in a complex case of TRD, improving quality of life, social functioning, and reducing suicidal ideation over a six-month follow-up.

**Disclosure:**

No significant relationships.

